# The effect of preoperative patient-reported anxiety on morbidity and mortality outcomes in patients undergoing major general surgery

**DOI:** 10.1038/s41598-022-10302-z

**Published:** 2022-04-15

**Authors:** Woubet Tefera Kassahun, Matthias Mehdorn, Tristan Cedric Wagner, Jonas Babel, Helge Danker, Ines Gockel

**Affiliations:** 1 Faculty of Medicine, Department of Visceral, Transplantation, Thoracic and Vascular Surgery, University Hospital of Leipzig, Liebigstr. 20, 04103 Leipzig, Germany; 2 Department of Medical Psychology and Sociology, University of Leipzig, Leipzig, Germany

**Keywords:** Medical research, Outcomes research

## Abstract

Excessive levels of anxiety may negatively influence treatment outcomes and likely increase patient suffering. We designed a prospective observational study to assess whether preoperative patient-reported anxiety affects major general surgery outcomes. We prospectively administered the State-Trait Anxiety Inventory (STAI) to measure preoperative anxiety in patients awaiting major general surgical procedures. Patients were grouped by STAI scores according to established cutoffs: no anxiety (STAI < 40) and anxiety (STAI ≥ 40). Four hundred patients completed the questionnaires and underwent surgery, with an average interval from questionnaire completion to surgery of 4 days. Applying a state anxiety (STAI-S) score ≥ 40 as a reference point, the prevalence of patient-reported anxiety was 60.5% (241 of 400). The mean STAI-S score for these patients was 50.48 ± 7.77. The mean age of the entire cohort was 58.5 ± 14.12 years. The majority of participants were male (53.8%). The distribution of sex by anxiety status showed that 53.5% of women and 46.5% of men had anxiety (*p* = 0.003). In the entire cohort, postoperative complications occurred in 23.9% and 28.6% of the no anxiety and anxiety groups, respectively. The difference was nonsignificant. In a subgroup of patients who underwent high-risk complex procedures (*N* = 221), however, postoperative complications occurred in 31.4% and 45.2% of the no anxiety and anxiety groups, respectively. This difference was significant at *p* = 0.004. Of the patients who were anxious, 3.3% (8 of 241) died during hospitalization following surgery, compared with 4.4% of the patients (7 of 159) who were not anxious (*p* = 0.577). In the multivariable analysis adjusted for covariates and based on the results of subgroup analysis, preoperative anxiety assessed by the STAIS score was associated with morbidity (OR 2.12, CI 1.14–3.96; *p* = 0.018) but not mortality. The majority of enrolled patients in this study were classified as having high- to very high-level preoperative clinical anxiety, and we found a significant quantitative effect of patient-reported anxiety on morbidity but not mortality after surgery.

## Introduction

The majority of patients awaiting surgery are anxious^1^, and some degree of anxiety is a natural reaction to the unpredictable and potentially threatening situations typical of the preoperative period^2^. However, an excessive level of anxiety may have a negative impact on treatment outcomes, and this has been demonstrated in patients in different medical settings.

Armfield et al.^3^ demonstrated that dental fear is associated with more decayed and missing teeth but fewer filled teeth. Moreover, people with higher dental fear experience significantly more caries.

It is well established that increased levels of preoperative anxiety are associated with worse postoperative pain, extended hospital stays and frequent readmission^4–6^.

The negative influence of patient-reported anxiety on outcomes has also been demonstrated in patients undergoing cardiac surgery.

Székely et al.^7^ studied patients who underwent cardiac surgery and found an independent association between anxiety and mortality.

Preoperative anxiety was evaluated in a cohort of older patients undergoing cardiac surgery by Williams et al.^5^, and significantly higher mortality was observed among older patients who experienced high levels of preoperative anxiety.

However, in the present era of modern and minimally invasive surgery, the association between preoperative patient-reported anxiety and adverse outcomes following major general surgical procedures has not been extensively studied. Thus, a study on this topic seemed useful. We therefore examined the association between patient-reported anxiety and postoperative morbidity and mortality. Our hypothesis was that given previous experience in other medical settings, patient-reported anxiety would negatively influence outcomes following major general surgical procedures. Our objective was to obtain more detailed information on the role of patient-reported anxiety in surgical outcomes for this patient population.

## Methods

Adult patients undergoing elective major general surgical procedures were included in this prospective observational study. The study was conducted from September 2018 through April 2021 at the Department of Visceral, Transplantation, Thoracic and Vascular Surgery of the University Hospital of Leipzig.

The majority of the subject pool comprised patients who underwent major and complex procedures, such as gastroesophageal, colorectal, liver and pancreatic resection.

The inclusion criteria were a minimum age of 18 years, provision of informed consent, and a scheduled general surgical procedure for any diagnosis.

The exclusion criteria were age under 18 years, outpatient surgical procedures, emergent surgical procedures, cancellation of the scheduled procedure, severe psychiatric, communication and cognitive disorders, active history of alcohol and drug abuse, inability to speak German, and noncompliance.

The patients were evaluated with the State-Trait Anxiety Inventory (STAI) and the Amsterdam preoperative anxiety and information scale (APAIS), two previously validated questionnaires for assessing preoperative anxiety levels in patients.

The STAI was developed by Spielberger^8^, translated into German and validated by Laux et al.^9^ It consists of a total of 40 items, 20 of which describe trait anxiety (STAI-T), and 20 of which describe state anxiety (STAI-S); patients record which one of four descriptors best indicates the degree of their emotion (score 1–4; minimum score 20, maximum score 80). A score ≥ 40 on either subscale is considered to indicate cases of high-level anxiety, and a score of 50 or higher suggests a very high level of anxiety in the German population^10^.

While STAI-T is a personality disposition that is relatively stable, STAI-S is situational and is related to a specific stressful event such as surgery. As such, it may differ depending on the stress experienced regarding the particular situation. STAI-S is therefore recommended for measuring preoperative anxiety^11^ and has been used in several studies^10,12^. State and trait anxiety were highly correlated (r = 0.67, *P* =  < 0.0001) in this study. We performed the analysis in this study based on STAI-S, as this is presumably a better reflection of current anxiety and a widely accepted measure in medical research. For the purpose of this study, we defined a STAI-S score ≥ 40 as indicative of clinical anxiety.

In addition, to determine more about the specific situation over which the patient was experiencing anxiety, i.e., to assess anxiety about anesthesia and anxiety about surgery separately, we used the German version^13^ of the six-question APAIS.

The APAIS is a self-reported six-item questionnaire that has been validated for assessing preoperative anxiety^14^. The items are rated on a five-point Likert scale, where a value of 1 indicates “not at all”, and a value of 5 indicates “extremely anxious”. It was divided into subscales to separate anxiety about anesthesia (items 1 and 2), anxiety about surgery (items 4 and 5) and the need for information (items 3 and 6), resulting in a potential range of scores from 2 to 10 for each subscale. A higher value reflects a higher anxiety level (the sum of questions 1, 2, 4, and 5; cutoff value ≥ 11 for clinical anxiety) as well as a greater need for information (the sum of questions 3 and 6). The anxiety items of the APAIS correlate with those of the state anxiety scale, with r = 0.60, *P* < 0001.

The items of the STAI and APAIS have been described in detail and published previously^8,14^.

After patients registered at the registration desk in the central surgical patient management wing, eligible patients were given a letter of explanation of the study by a member of the research team. Patients were informed that they were under no obligation to participate in the study. After informed consent was obtained for surgery, informed consent for anesthesia and participation in the present study was obtained, patients were given a copy of the STAI and APAIS, in that order, that they completed while in the waiting room or at ward. Patients were also asked to report their level of education, occupation, employment and marital status and number of children, if any. The treating physicians were unaware of the results of the questionnaires to avoid the results influencing perioperative care.

## Clinical and demographic characteristics

A review and analysis of the data for all patients who had been entered in a prospective data registry that included patient and disease characteristics and outcomes was conducted. Based on the principal operative procedure, all elective operations were categorized as involving the upper gastrointestinal (GI) tract, lower GI tract, hepato-bilio-pancreatic (HBP), thoracic-wall-pulmonary, and miscellaneous systems. Only patients whose procedure warranted more than an overnight stay were selected.

Patient characteristics, disease entity and surgical variables that might have been potential confounders of postoperative outcomes were examined.

The main outcome measure was the incidence of complications or mortality, defined as hospital death from any cause occurring after surgery during hospitalization, including those patients who returned to the hospital shortly after discharge for complications directly related to the index procedure.

The severity of medical conditions at the time of surgery was evaluated using the American Society of Anesthesiologists (ASA) Physical Status classification^15^. The Clavien-Dindo (CD) classification of surgical complications^16^ was used to classify surgical complications. In addition, according to the CD classification at discharge, the comprehensive complication index (CCI)^17^ was calculated for each patient to evaluate the true overall morbidity burden of a procedure.

Surgical procedures are classified as minor and major depending on the type of anesthesia and the estimated duration of operative time. For the purpose of this study, procedures with an estimated operative time ≥ 90 min that were performed under general anesthesia (100%) were considered major procedures.

Patients were grouped according to STAI scores using established cutoffs: no anxiety (STAI < 40) and anxiety (STAI ≥ 40). Baseline patient characteristics, comorbidities and operative variables were compared across the no anxiety and anxiety groups. In addition, we independently analyzed the results of a subgroup of patients with known complex high-risk procedures mainly due to cancer, as we did for the entire study cohort.

Statistical analysis was performed using SPSS software version 27 for Windows (IBM Corporation, USA). Descriptive statistics were calculated to assess the distribution of patients, procedures, comorbidities, morbidity and mortality by group. Univariate statistical comparisons between groups were performed using the chi-square test, or Student’s t-test, as appropriate. To control for potential confounders, we first examined the univariate relation between preoperative risk factors and outcome variables. We then performed multivariable analysis using logistic regression modeling. Because the number of documented risk factors (comorbidities and postoperative complications) in patients undergoing general surgery in this study is large (> 15, Supplemental tables 2 and 3), entering all covariates in the model would have resulted in model instability and overfitting. Therefore, we used the ASA as the simplest and most widely used system to provide preoperative risk assessment (illness severity score)^15^ and, based on the Clavien-Dindo classification of complications^16^, the CCI score as a reflection of the overall burden of postoperative complications^17^ to adjust for the surgical risk associated with traditional pre- and postoperative clinical risk factors. STAI-S, as an indicator of surgical risk and a primary independent variable was entered into a multivariate logistic regression analysis, with outcome variables as dependent variables and the risk factors as independent variables, to test for significant effects while simultaneously adjusting for multiple factors. Model fit was evaluated using the area under the receiver operating characteristic curve (AUROC)^18^. The results were expressed as arithmetic means ± standard deviations, medians with ranges (Rs) or frequencies with percentages. Relative risks (RRs) or Odds ratios (ORs) and 95% confidence intervals (CIs) were estimated. Correlations, where appropriate, were calculated using Pearsonʼs correlation coefficient. All statistical tests were two-tailed, and *p* values ≤ 0.05 were considered statistically significant.

This study was approved by the Institutional Review Board of the medical faculty of the University of Leipzig in Leipzig, Germany, registration number: 102/18-ek. Formal written informed consent was obtained from the participants. We confirm that all methods are carried out in accordance with the relevant guidelines and regulations of the ethics committee.

The study is registered in the German register for clinical trials at https://www.drks.de/ui_data_web/DrksUI.html with the unique identifying number DRKS00018798.

The paper was written in compliance with the standards set forth by the Strengthening the Reporting of Observational Studies in Epidemiology (STROBE) statement^19^.

## Results

### Patients’ characteristics

**Figure 1:**
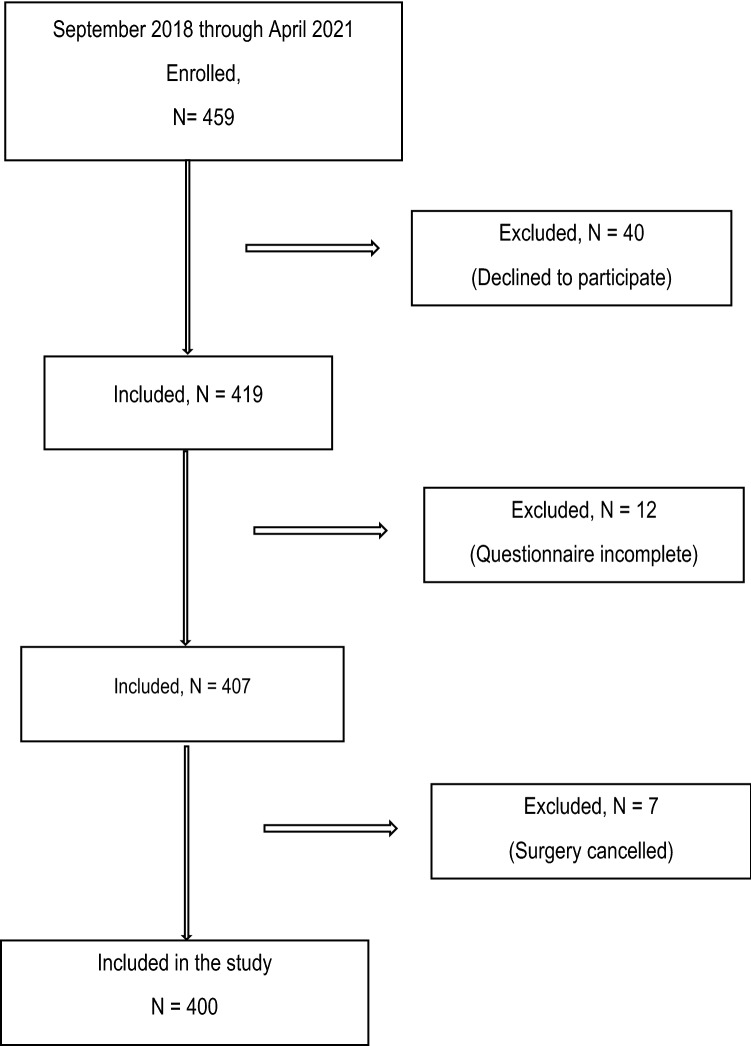
Flow diagram of patient selection.

The flow of patients through the present study is depicted in Fig. 1. Four hundred patients completed the questionnaires and underwent surgery, with an average interval from questionnaire completion to surgery of 4 days. Ideally, patients should be operated on the day after admission. In real-world clinical practice, however, it does not always work in this timely manner, particularly when the procedure is complex, and the diagnosis is cancer. Some of the reasons were, for example, a missed diagnostic procedure, which has relevance for surgery that cannot be organized overnight. Another reason was postponing the procedure so that it would take place later than originally planned because the operation theater was filled to capacity due to emergency procedures or organ transplantation that could not be postponed. Nevertheless, with an average interval from questionnaire completion to surgery of 4 days, we are within the limit of 1 week that was reported in previous studies^5,7,14,20,21^.

With an STAI-S score of ≥ 40 as a reference point, the prevalence of patient-reported anxiety was 60.5% (241 of 400). Of those with anxiety, 125 (51.9%) had a high level of anxiety (STAI-S 40 to 49), and 116 (48.1%) had a very high level of anxiety (STAI-S ≥ 50).

To assess the components of anxiety (anxiety about anesthesia and anxiety about surgery) separately, we used the six-question APAIS. Thirty-four percent of the anxious patients had anxiety related to anesthesia (mean APAIS score = 7.29 ± 1.32 out of 10), and 71.8% had anxiety related to surgery (mean APAIS score = 7.55 ± 1.43 out of 10).

The patients´ baseline characteristics are shown by anxiety status in Table 1.

**Table 1:** Baseline patient characteristics (*N* = 400).

Variable	No anxiety	Anxiety	*p* value
	(STAI < 40; *n* = 159)	(STAI ≥ 40; *n* = 241)	
**STAI-S score, mean ± SD**	**32.69 ± 5.04**	**50.48 ± 7.77**	** < 0.001**
QC, days, mean ± SD	3.48 ± 2.59	3.54 ± 2.64	0.907
Sex			
**Female**	**56 (35.2)**	**129 (53.5)**	**0.003**
Education			
College/university	46 (28.9)	75 (31.1)	0.641
Employment			
Full-time	100 (62.9)	155 (64.3)	0.772
Marital status			
Married	104 (65.4)	151 (62.7)	0.575
Children			
Yes	76 (47.8)	107 (44.4)	0.504
Comorbidity profile			
Age, years, mean ± SD	59.28 ± 13.31	57.90 ± 14.65	0.340
Age, years, ≥ 70	38 (23.9)	54 (22.4)	0.728
BMI, mean ± SD	29.73 ± 9.24	29.21 ± 10.34	0.604
COCs	140 (88.1)	214 (88.8)	0.819
COCs pp, mean ± SD	3.71 ± 2.38	3.56 ± 2.10	0.539
ASA ≥ 3	49 (30.8)	86 (35.7)	0.314
Hypertension	90 (56.6)	140 (58.1)	0.768
AF	22 (13.8)	28 (11.6)	0.512
CHF	14 (8.8)	11 (4.6)	0.086
CAD	12 (7.5)	22 (9.1)	0.579
PAD	11 (6.9)	20 (8.3)	0.613
COPD	26 (16.4)	28 (11.6)	0.175
Diabetes mellitus	41 (25.8)	56 (23.2)	0.560
Chronic renal failure	14 (8.8)	23 (9.5)	0.803
History of malignancy	42 (26.4)	66 (27.4)	0.831
History of previous surgery	97 (61.0)	134 (55.6)	0.284

STAI, State-Trait Anxiety inventory (STAI-S = state anxiety); QC, questionnaire completion to surgery; BMI, body mass index in kg/m^2^; SD, standard deviation; AF, atrial fibrillation; CHF, chronic heart failure; CAD, coronary artery disease; PAD, peripheral artery disease; COPD, chronic obstructive pulmonary disease; ASA, the American society of anesthesiologists physical status classification; COCs, comorbid conditions; pp, per patient. Numbers in bracket indicate values presented in *n* (%) by group unless noted otherwise. *P* values represent the difference between the two groups.

Significant values are in bold.

The mean score on the STAI was 32.69 ± 5.04 vs. 50.48 ± 7.77 for the no anxiety and anxiety groups, respectively. This difference was statistically significant (*p* < 0.001).

The mean age of the entire cohort was 58.5 ± 14.1 years, and the majority of participants were male (53.8%).

The distribution of sex by anxiety status showed that 53.5% of the women (129 of 185) had anxiety compared to 46.5% of the men (112 of 215), and there were more women in the anxiety group (129, 53.5%) than in the no anxiety group (56, 35.2%). This difference was statistically significant (*p* = 0.003). After adjusting for highly suspected risk factors, including age, employment status, and social support, female sex remained the only independent predictor of anxiety (OR 2.15, 95% CI 1.41–3.28, *p* = 0004).

The anxiety group did not differ significantly from the no anxiety group with respect to mean age, body mass index (BMI), or coexisting conditions, including previous surgery, history of malignancy, level of education, employment status, and marital status.

### Surgical variables

Table 2 shows the surgical variables by group.

**Table 2:** Surgical variables (*N* = 400).

	No anxiety	Anxiety	*p* value
	(STAI < 40; *n* = 159)	(STAI ≥ 40; *n* = 241)	
Disease entity			
Malignant	94 (59.1	136 (56.4)	0.595
Surgical accesses			
MI-Laparoscopic	60 (38.0)	95 (39.4)	0.832
MI-Robotic	21 (13.2)	25 (10.4)	0.385
Open	78 (49.1)	121 (50.2)	0.822
Procedure category			
**Upper GI**	**67 (42.1)**	**74 (30.7)**	**0.032**
Lower GI	37 (23.3)	54 (22.4)	0.859
**HBP**	**29 (18.2)**	**75 (31.1)**	**0.001**
Thoracic wall-pulmonary	10 (6.3)	13 (5.4)	0.872
Miscellaneous	16 (10.1)	25 (10.4)	0.961
Operative time, min, mean ± SD	250.8 ± 154.2	235.6 ± 136.4	0.300

MI, minimally invasive; GI, Gastrointestinal tract; HBP, Hepato-Bilio-Pancreatic; min, minutes. Numbers in bracket indicate values presented in *n* (%).

Significant values are in bold.

In the overall study group, there were 141 upper GI cases (35.3%), 91 lower GI cases (22.6%), 104 HBP cases (26.0%), 23 thoracic wall pulmonary cases (5.6%), and 41 cases (10.3%) categorized as other general surgeries.

The two groups were not markedly different in disease entity, surgical access, operative time or procedure type, except for upper GI and HBP procedures. The anxiety group underwent fewer upper GI procedures (30.7% vs. 42.1%, *p* = 0.032) but more HBP procedures (31.1% vs. 18.2%, *p* = 0.001) than the no anxiety group. Details of index procedures are shown in supplemental table 1.

Thus, the two groups in this study were more homogeneous in terms of many of the evaluated variables.

### Clinical outcomes

The clinical outcomes are listed by group in Table 3.

**Table 3:** Outcomes (*N* = 400).

Variable	No anxiety	Anxiety	*p* value
	(STAI < 40; *n* = 159)	(STAI ≥ 40; *n* = 241)	
Complications overall	38 (23.9)	69 (28.6)	0.295
Complications pp, mean ± SD	2.59 ± 2.06	2.36 ± 1.94	0.572
CCI, mean ± S	23.88 ± 24.17	23.83 ± 22.07	0.983
Hemorrhage	11 (6.9)	17 (7.1)	0.958
Surgical site infection	21 (13.2)	35 (14.5)	0.711
Anastomotic leak	14 (8.8)	30 (12.4)	0.254
Pneumonia	14 (8.8)	30 (12.4)	0.254
Thromboembolic events	7 (4.4)	11 (4.6)	0.939
Liver failure	6 (3.8)	8 (3.3)	0.809
Acute renal failure	9 (5.7)	11 (4.6)	0.623
Reoperation	11 (6.9)	17 (7.1)	0.958
Reoperation pp, mean ± SD	1.73 ± 1.01	2.11 ± 2.17	0.587
ICU	81 (50.9)	131 (54.4)	0.775
ICU-LOS, days, mean ± SD	4.59 ± 9.44	3.55 ± 8.51	0.096
ICU-LOS, days, median (R)	1 (1–67)	1 (1–89)	
Readmission	70 (44.9)	97 (40.8)	0.419
Mortality	7 (4.4)	8 (3.3)	0.577
LOS, days, mean ± SD	14.49 ± 15.90	15.87 ± 15.02	0.381
LOS, days, median (R)	9 (2–98)	10 (3–97)	
LOS > 14 days	49 (30.8)	87 (36.1)	0.275

pp, per patient; surgical site infection is defined as being contained within the skin or subcutaneous tissue (superficial), or involving the muscle and /or fascia (deep); CCI, the comprehensive complication index; ICU, intensive care unit requirement; LOS, length of hospital stay defined as the time from the date of the initial admission to the date of discharge, transfer to external services, or death, which ever came first. Numbers in bracket indicate values presented in *n* (%).

In the overall cohort (*N* = 400), postoperative complications occurred in 23.9% of the no anxiety group (38 of 159) and 28.6% of the anxiety group (69 of 241).

In the unadjusted analysis with the STAI-S score as a dichotomous variable (score ≥ 40 signifying anxiety vs. < 40 signifying no anxiety), patients with anxiety were at slightly increased risk of postoperative complications compared with those who were not anxious (RR 1.28, 95% CI 0.81–2.02, *p* = 0.295). However, this difference did not reach statistical significance.

Age ≥ 70 years (RR 1.61, 95% CI 1.12–2.31, *p* = 0.012), cancer diagnosis (RR 1.56, 95% CI 1.35–1.83, *p* =  < 0.001), open surgery (RR 2.04, 95% CI 1.72–2.43, *p* =  < 0.0001), esophagogastric surgery (RR 1.94 95% CI 1.1.18–3.20, *p* = 0.009), HBP surgery (RR 2.14, 95% CI 1.56–2.93, *p* =  < 0.001), and longer operative time (RR 1.13, 95% CI 1.07–1.20) were significantly associated with an increased risk of complications in the unadjusted model. Supplemental Table 2.

After adjusting for covariates, open surgery and HBP procedures remained significant predictors of morbidity, as shown in Table 4.

**Table 4:** Multivariate analysis: predictors of postoperative complications or mortality after adjusting for risk factors (entire cohort, *N* = 400).

Risk factor	Postoperative complications	
	OR (95% CI)	*P*
Age	1.23 (0.71–2.19)	0.445
Diagnosis of cancer	1.75 (0.95–3.21)	0.072
**Open surgery**	**4.05 (2.29–7.16)**	** < 0.001**
**HBP-Surgery**	**2.21 (1.23–3.94)**	**0.008**
STAI_S score ≥ 40	1.29 (0.77–2.16)	0.330
In-hospital mortality		
Age	2.78 (0.94–8.18)	0.064
ASA	2.80 (0.93–8.39)	0.066
**CCI**	**14.02 (1.80–109.12)**	**0.012**
STAI_S score ≥ 40	0.64 (0.22–1.9)	0.423

Each of the risk factors represents a significant univariate predictor of complications or mortality. OR, odds ratio CI, confidence interval; ASA, the American society of anesthesiologists physical status classification, CCI, the comprehensive complication index.

Significant values are in bold.

The incidence of specific complications did not differ significantly between groups, nor did the need for intensive care unit (ICU), readmission rate, or length of hospital stay.

Overall, 167 patients (70 patients [44.9%] in the no anxiety group and 97 patients [40.8%] in the anxiety group) were readmitted after discharge for any reason. The percentage of patients who were readmitted for complications related to the index procedure was not significantly different between the anxiety and no anxiety groups (12.5% vs. 15.1%, *p* = 0.584).

Overall, 15 patients died after surgery, yielding an overall in-hospital mortality rate of 3.8%. Analysis of the mortality rate by group indicated that 3.3% of the patients (8 of 241) who were anxious died during hospitalization following surgery, compared with 4.4% of the patients (7 of 159) who were not anxious (*p* = 0.577). Thus, the mortality rate of the anxiety group was similar to that of the no anxiety group.

In multivariable analysis adjusted for covariates, preoperative patient-reported anxiety was not associated with mortality. The point estimates and CIs for the multivariate analysis are shown in Table 4.

### Subgroup analysis

Because the sample size of each prespecified general surgical procedure was small for meaningful statistical results, we reanalyzed a subgroup of patients (*N* = 221, 55.3% of the study population) with the most complex surgical procedures mainly due to cancer (77.4%). This included those patients who underwent esophageal resection, including gastrectomy (*n* = 51), colorectal resection (*n* = 81) and hepatopancreatic resection (*n* = 89). These procedures are high-risk surgical procedures that require substantial surgical skill. Patients who undergo these procedures are at high risk of postoperative complications and death^22–25^. In our study, all 15 deaths and 82.2% (88 of 107) of morbidity cases (at least one postoperative complication) occurred in this subgroup of patients. Therefore, these procedures were chosen as representative of complex procedures for the purpose of subgroup analysis in this study. The results are depicted in Table 5. See also Supplemental table 4 for individual specific procedures.

**Table 5:** Baseline patient characteristics and main outcomes for subgroup of patients who underwent 3 specific complex procedures (*N* = 221).

Variable	No anxiety	Anxiety	*p* value
	(STAI < 40; *n* = 86)	(STAI ≥ 40; *n* = 135)	
**STAI-S score, mean ± SD**	**32.98 ± 5.18**	**50.79 ± 8.31**	** < 0.001**
**Female**	**24 (27.9)**	**64 (47.4)**	**0.004**
Age, years, mean ± SD	63.62 ± 11.55	61.24 ± 14.09	0.192
BMI, mean ± SD	26.29 ± 4.88	26.56 ± 5.61	0.712
COCs	75 (87.2)	120 (88.9)	0.706
ASA ≥ 3	26 (30.2)	50 (37.0)	0.299
Open surgery	52 (60.5)	76 (56.3)	0.541
Cancer	71 (82.6)	100 (74.1)	0.142
OT, min, mean ± SD	333.63 ± 143.35	305.60 ± 122.66	0.123
ICU	65 (75.6)	102 (75.6)	0.997
**Morbidity**	**27 (31.4)**	**61 (45.2)**	**0.004**
Mortality	7 (8.1)	8 (5.9)	0.524
LOS, days, mean ± SD	21.14 ± 19.18	20.30 ± 15.86	0.723

STAI, State-Trait Anxiety inventory (STAI-S = state anxiety); BMI, body mass index in kg/m^2^; SD, standard deviation; ASA, the American society of anesthesiologists physical status classification; COCs, comorbid conditions; pp, per patient; OT, operative time; ICU, intensive care unit required; LOS, hospital length of stay. Numbers in bracket indicate values presented in *n* (%) by group unless noted otherwise. *P* values represent the difference between the two groups.

Significant values are in bold.

The prevalence of patient-reported anxiety in this subgroup of patients was 61.1% (135 of 221). As depicted in Table 5, the anxiety group did not differ from the no anxiety group with respect to age, BMI, coexisting conditions, disease entity, surgical access, operative time and other variables. As in the overall study population, the anxious (*N* = 135) and nonanxious (*N* = 86) patients were homogenous in terms of many of the evaluated variables except their appropriate STAIS scores and sex distribution, and there were no sex-related differences regarding the occurrence of postoperative complications.

The cumulative percentage of complications in both groups with at least one postoperative complication was 39.8% (88 of 221) and was significantly higher for patients in the anxiety group than for patients in the no anxiety group (45.2% versus 31.4%, *p* = 0.004).

In this subgroup of patients who received the most complex surgical procedures, patients who had preoperative anxiety were at significantly increased risk for developing at least one postoperative complication (OR 2.12, CI 1.14–3.96; *p* = 0.018), see Table 6.

**Table 6:** Multivariate analysis: Predictors of postoperative complications after adjusting for risk factors (subgroup of patients with complex procedures, *N* = 221).

Risk factor	Postoperative complications	
	OR (95% CI)	*P*
**STAIS ≥ 40**	**2.12 (1.14–3.96)**	**0.018**
**Open surgical access**	**3.59 (1.85–6.97)**	** < 0.001**
**OT, min, > 180**	**2.93 (1.05–8.20)**	**0.040**
BMI ≥ 30	1.84 (0.89–3.80)	0.099

Adjusted for STAIS, age, BMI, ASA, open surgical access, cancer diagnosis, and longer operative time. OR, odds ratio CI, confidence interval; ASA, the American society of anesthesiologists physical status classification.

Significant values are in bold.

The overall in-hospital mortality rate in both groups was 6.8% (15 of 221), and there was no significant difference between groups. Furthermore, preoperative patient-reported anxiety had no significant effect on ICU requirement or the length of hospital and ICU stays in this subgroup of patients.

In summary, we identified significantly increased postoperative morbidity in anxious patients who received the most complex surgical procedures compared to nonanxious patients.

## Discussion

In this study, we set out to determine the effect of patient-reported anxiety on morbidity and in-hospital mortality in a cohort of general surgery patients. For the entire study cohort, the results demonstrated that patient-reported anxiety assessed by the STAI-S before surgery does not predict an increased risk of morbidity or mortality. Compared with those who were not anxious, patients with anxiety had a slightly but not significantly increased risk of overall complications postoperatively. The incidence of specific complications did not differ significantly between groups, nor did the need for ICU admission, readmission rate, or length of hospital stay. The mortality rate of the anxiety group was similar to that of the no anxiety group.

However, in a subgroup analysis, our findings indicate that preoperative patient-reported anxiety was associated with morbidity among a subgroup of patients undergoing complex general surgical procedures, based on the presence of significant differences in the occurrence of at least one postoperative complication.

This difference cannot be explained by differences in baseline patient characteristics, including social status, lifestyle and coexisting conditions, as these were not significantly different between the anxiety and no anxiety groups. The results of the subgroup analysis in our study are in agreement with the results of previous studies that demonstrated adverse outcomes in anxious patients in different medical settings^3–7,21,26^.

The relationship between preoperative patient-reported anxiety and adverse patient outcomes has not been clearly defined. However, previous studies have proposed several mechanisms to explain the negative impact of anxiety on outcomes^1,27–30^. These mechanisms include excessive cortisol production with insulin resistance and sympathetic and vagal disturbances. The stress hormone cortisol, for example, regulates the body´s stress response. However, assuming that the patient may continue to experience anxiety beyond the operation day while in treatment, elevated cortisol levels as a response of excessive anxiety may suppress the immune system precipitating postoperative infectious complications. The most common complications in our study were infectious in nature, and their prevalence was higher in anxious patients. These included surgical site infection (23.7% vs. 18.6%), anastomotic leaks (20% vs. 15.1%), and pulmonary complications (16.3% vs. 9.4%).

On the other hand, in contrast to the results of previous studies on cardiac surgery patients^5,7^, overall and in a subgroup analysis, our study demonstrated a poor correlation between preoperative patient-reported anxiety and in-hospital mortality. However, given the small sample sizes in those studies, adequate statistical power to assess the effect of anxiety on mortality outcomes is lacking.

In contrast, our study cohort had a sizable number of patients and evaluated the effect of preoperative anxiety on outcomes after major general surgery. The majority (> 60%) of the patients were classified as having clinical anxiety. After controlling for other covariates in our study, however, there was no significant association between patient-reported anxiety and in-hospital mortality.

The reasons for the poor association are not entirely clear. However, as outcomes improve over time, the risk of mortality associated with several risk variables, such as preoperative anxiety level, may change substantially. This means that as outcomes improve, certain risk factors may lose their importance as predictors of mortality outcomes.

In recent decades, the introduction and adoption of minimally invasive surgery (MIS) techniques in various surgical specialties, including general surgery, has led to significant improvements in postoperative patient care and outcomes^31,32^. More than 50% of the studied patients underwent MIS, including robotic-assisted surgery. Among 15 deaths overall, there were only 3 deaths (mortality rate, < 1%) among patients operated on using MIS techniques, and none of those mortality cases were in the anxiety group. In comparison, the overall morbidity or mortality for open surgery in this study was more than threefold higher, and all of the mortality cases in the anxiety group were operated on using open surgical techniques. Therefore, the poor association between preoperative patient-reported anxiety and in-hospital mortality may be a reflection on modern surgical techniques. On the one hand, mortality outcomes improved partly because of improved surgical techniques; on the other hand, although anticipated, preoperative information about the type of surgical access (informed consent) did not affect (reduce) the number of patients with high preoperative anxiety levels in this study. Almost half of the anxious patients underwent MIS. Thus, the findings of this study suggest that regarding the prediction of mortality outcomes after surgery in an era of increasing use of minimally invasive techniques, traditional clinical factors such as the burden of overall complications, which is related to the type of surgical procedure and the type of surgical access, may increase the risk of mortality more than emotional tension. Open surgery, HBP procedures, and the CCI score as a reflection of the burden of postoperative complications independently predicted morbidity or mortality in the overall cohort in this study, but preoperative patient-reported anxiety appears to add little information toward predicting the risk of in-hospital mortality after surgery.

According to the scores on the surgical components of the APAIS, more than 71% of the anxious patients in this study referred their anxiety to surgery (score ≥ 7). This may be due to an exaggerated perception of surgical risk. Overestimation of the risk of perioperative mortality is common in patients undergoing general surgery and is associated with preoperative anxiety^33^. However, because of the diffuse nature of preoperative anxiety, the APAIS did not distinguish clearly between anxiety related to surgery and that related to anesthesia^2,14^. As such, this analysis was performed merely with regard to the components of anxiety and not with the intent of demonstrating the cause of anxiety. Therefore, the results should be interpreted with caution.

Similarly, according to the scores on the information scale of the APAIS, 81.3% of the anxious patients scored ≥ 5, indicating a positive attitude toward receiving more information, which is in agreement with the work of other researchers^14^.

Thus, the recognition of preoperative anxiety and understanding its personal meaning among general surgery patients is clinically important because in addition to its negative effect on postoperative morbidity, it is a psychological discomfort that likely increases patient suffering. This means that along with the legal obligation of obtaining informed consent, miscellaneous interventions could be instituted to address this discomfort and modify patient attitudes.

For example, more personal attention and additional information regarding the procedure, personal experience and previous outcomes from the surgeon performing the procedure as well as stress management and psychological intervention services, if available, should be integrated early rather than late in the preoperative course.

Effective and skilled communication may help patients realistically appraise the relationship between surgery and behavior, as inadequate information transmission during an interaction has an anxiety-provoking effect^34^.

Another finding in this analysis was the distribution of sex by anxiety status. A total of 53.5% of the women had anxiety compared to 46.5% of the men, and there were significantly more women in the anxiety group than in the no anxiety group. Female sex predicted anxiety independently. The explanation behind the sex-related disparity in preoperative anxiety is not entirely clear. However, as anxiety was self-reported in this study, the sex-related differences probably reflect the willingness of female patients to admit anxiety.

In this regard, the results of other studies are mixed. In some studies, more women had anxiety than men^1,10^, yet in others, either there were no gender-related differences or fewer women than men had anxiety^2,5,13,35^.

Regarding the relation between anxiety and gender in the general public, recent population-based studies show that women are more likely to develop anxiety than men^36,37^. Therefore, based on recent data, the reported sex difference in the present study seems not to be unique to anxiety in surgical patients.

### Study limitations

There are some important limitations of this work. First, this study was an observational study from which causality cannot be inferred, and the influence of other confounders on the presented results cannot be excluded. For example, chronic pain may be one of these factors, but was not evaluated in this study. The major outcome measure in our study was the occurrence of postoperative complications or the in-hospital mortality rate after general surgical procedures. This included all complications and deaths after surgery within the first hospital stay, without the patient being discharged from the hospital. We undertook a more focused approach and examined the effect of preoperative patient-reported anxiety on these outcome measures and not the association between chronic pain and preoperative anxiety or outcomes.

Second, the best methods for determining preoperative patient-reported anxiety are debatable. However, the two measures used in this study, STAI-S and the APAIS, are simple direct psychological measures and capture the essence of the subjective experience. Patients are questioned regarding their feelings about surgery, and their responses are combined to objectively quantify anxiety in the preoperative period. As the questions directly address subjective feelings, they usually provide a good estimate of how anxious patients truly are.

Third, although our original cohort consisted of more than 450 patients, those whose surgery was cancelled and those who refused to participate in the study or complete the questionnaire were not enrolled. Because not all operated patients in the study time frame were included in this study for a variety of reasons, ranging from the medical unfitness of patients to participate to the unavailability of research personnel, participants in this study might not be fully representative of the operated patient population.

Finally, this study involved patients from a single center, and because of this, our results may not be broadly generalizable. Therefore, the findings remain to be validated in a larger multi-institutional data set.

Despite these limitations, we feel that this prospective study is of clinical importance because it addresses the important issue of preoperative emotional tension and provides pertinent data on the role of patient-reported anxiety in surgical outcomes in general surgery patients. Our observation should encourage the recognition of anxiety among surgical patients before complex surgical procedures and promote collaboration among surgeons and psychology services to help the patient manage his anxiety and adjust it to an appropriate level, which may by extension reduce the rate of adverse patient outcomes.

## Conclusions

In conclusion, the majority of the patients in the present study were classified as having high- to very high-level preoperative clinical anxiety. In a subgroup analysis, our findings indicate that preoperative patient-reported anxiety was associated with morbidity but not mortality among a subgroup of patients undergoing complex general surgical procedures, based on the presence of significant differences in the occurrence of at least one postoperative complication. Excessive preoperative anxiety levels may indicate an exaggerated perception of surgical risk and preoccupation with surgery. On the other hand, it may also reflect processes of care that are not attuned to the needs of anxious patients, highlighting opportunities for improvements in care. While we cannot draw a firm conclusion on this point from our data, we feel that further studies examining the role of preoperative patient-reported anxiety in surgical outcomes in general surgery patients are warranted.

## Supplementary Information

Supplementary Information.

## Data Availability

The datasets generated and/or analyzed during the current study are not publicly available due to internal institutional restrictions but are available from the corresponding author on reasonable request and with the permission of the institution where the data were generated.
