# Supplementary material for: The effect of preoperative patient-reported anxiety on morbidity and mortality outcomes in patients undergoing major general surgery
Source: Sci Rep. 2022 Apr 15;12:6312. doi: 10.1038/s41598-022-10302-z (PMC9012824; doi:10.1038/s41598-022-10302-z)
Supplement: Supplementary file 1 — Supplementary Information. [file 41598_2022_10302_MOESM1_ESM.docx]

**Supplemental Table 1.** Index procedures (n = 400)

No anxiety Anxiety

Procedure (STAI < 40; n = 159) (STAI ≥ 40; n = 241)

Esophageal resection 19 (11.9) 19 (7.9)

Gastric resection of any type 17 (10.7) 21 (8.7)

Including gastrectomy

Gastric bypass surgery 17 (10.7) 14 (5.8)

Surgery of any type 14 (8.8) 20 (8.3)

for GERD and EA

Small bowel resection 4 (2.5) 5 (2.0)

Including ileostomy

Colorectal resection 33 (19.5) 49 (20.3)

Including colostomy

Liver resection 22 (13.8) 53 (22.0)

Pancreatic resection 7 (4.4) 22 (9.1)

Thoracic wall-pulmonary 10 (6.3) 13 (5.4)

Resection

Multivisceral resection 9 (5.7) 11 (4.6)

Nephrectomy 3 (1.9) 7 (2.9)

Hernia repair 3 (1.9) 2 (0.8)

Adrenalectomy 1 (0.6) 3 (1.2)

Splenectomy - 1 (0.4)

Thyroidectomy - 1 (0.4)

GERD, Gastroesophageal reflux disease; EA, Esophageal achalasia

**Supplemental Table 2.** Relative risk (RR) for postoperative complications for all patients (N = 400, Univariate analysis).

Risk factor RR (95% CI) p value

Age ≥ 70 years 1.61 (1.12-2.31) 0.012

ASA ≥ class 3 1.32 (0.99-1.76) 0.060

Female sex 0.83 (0.64-1.08) 0.142

BMI ≥ 30 0.94 (0.68-1.31) 0.716

Previous surgery 1.03 (0.85-1.24) 0.782

Hypertension 1.03 (0.86-1.25) 0.736

AF 1.54 (0.90-2.63) 0.114

PAD 1.12 (0.53-2.36) 0.765

COPD 1.49 (0.89-2.48) 0.132

Chronic renal failure 0.88 (0.43-1.80) 0.726

Diabetes mellitus 1.17 (0.80-1.70) 0.421

Diagnosis of cancer 1.56 (1.35-1.83) <0.001

Open surgery 2.04 (1.72-2.43) <0.001

Esophagogastric surgery 1.94 (1.18-3.20) 0.009

HBP-Surgery 2.14 (1.56-2.93) <0.001

Operative time ≥ 90 minutes 1.13 (1.07-1.20) 0.002

STAI-S ≥ 40 1.28 (0.81-2.02) 0.295

RR, relative risk; CI, confidence interval; ASA; American Society of Anesthesiologists Physical Status classification; HBP, Hepato-Bilio-Pancreatic ; BMI, body mass index in kg/m^2^ ; AF, atrial fibrillation; PAD, peripheral artery disease; COPD, chronic obstructive pulmonary disease; HBP, Hepato-Bilio-Pancreatic.

**Supplemental Table 3.** Relative risk (RR) for in-hospital mortality for all patients (N = 400, Univariate analysis).

Risk factor RR (95% CI) p-Value

Age ≥ 70 years 2.44 (1.47-4.07) 0.004

Open surgery 1.79 (1.43-2.24) 0.004

Diagnosis of cancer 1.66 (1.42-1.96) 0.004

AF 2.23 (0.92-5.39) 0.091

Esophageal resection 2.67 (1.25-5.73) 0.019

HBP-Surgery 2.12 (1.28-3.50) 0.015

ICU stay 1.82 (1.54-2.14) 0.001

Ventilator dependence 21.39 (11.03-41.46) < 0.001

Hemorrhage 8.56 (4.32-16.93) < 0.001

Surgical site infection 4.92 (3.00-8.04) < 0.001

Anastomotic leakage 7.55 (4.67-12.21) < 0.001

Pneumonia 6.60 (3.92-11.10) < 0.001

Thromboembolic events 12.83 (5.58-29.53) < 0.001

Acute renal failure 21.00 (10.28-42.89) < 0.001

Liver failure 64.17 (22.72-181.27) < 0.001

Reoperation 10.27 (5.43-19.42) < 0.001

LOS > 14 days 2.04 (1.39-3.00) 0.006

Operative time ≥ 120 minutes 1.29 (1.22-1.36) 0.039

OR, odds ratio; CI, confidence interval; HBP, Hepato-Bilio-Pancreatic; Ventilator dependence is defined as mechanical ventilation ≥ 24 hours; ICU, intensive care unit.

**Supplemental Table 4.** Main outcomes by groups for individual specific complex procedures (N = 221)

No anxiety Anxiety

Variable (STAI < 40, n = 86) (STAI ≥ 40, n = 135)

**Esophageal resection n = 28 (32.6) n = 23 (17.0)**

**Including gastrectomy**

Morbidity 10 (35.7) 12 (42.2)

Mortality 2 (7.1) 3 (13.0)

LOS, days, mean ± SD 27.50 ± 23.22 23.74 ± 11.17

**Colorectal resection n = 32 (37.2) n = 49 (36.3)**

Morbidity 6 (18.8) 15 (30.6)

Mortality 1 (3.1) 1 (2.0)

LOS, days, mean ± SD 16.09 ± 16.97 15.37 ± 11.87

**HBP resection n = 26 (30.2) n = 63 (46.7)**

Morbidity 10 (38.5) 33 (52.4)

Mortality 4 (15.4) 4 (26.3)

LOS, days, mean ± SD 20.50 ± 15.29 22.87 ± 18.98

STAI, State-Trait Anxiety inventory (STAI-S = state anxiety); HBP, hepato-bilio-pancreatic resection; LOS, hospital length of stay. Numbers in bracket indicate values presented in n (%) by group unless noted otherwise. P values represent the difference between the two groups
